# Potential of *in vivo* stress reporter models to reduce animal use and provide mechanistic insights in toxicity studies

**DOI:** 10.12688/f1000research.123077.2

**Published:** 2023-08-10

**Authors:** Francisco Iñesta Vaquera, Febe Ferro, Michael McMahon, Colin J. Henderson, C. Roland Wolf

**Affiliations:** 1 Systems and Cellular Medicine, University of Dundee, Dundee, DD1 9SY, UK

**Keywords:** Risk assessment, alternative in vivo methods, chemical toxicity, early biomarkers, in vivo toxicology, mechanistic toxicology, oxidative stress, DNA damage.

## Abstract

Chemical risk assessment ensures protection from the toxic effects of drugs and manmade chemicals. To comply with regulatory guidance, studies in complex organisms are required, as well as mechanistic studies to establish the relevance of any toxicities observed to man. Although *in vitro* toxicity models are improving, *in vivo* studies remain central to this process. Such studies are invariably time-consuming and often involve large numbers of animals. New regulatory frameworks recommend the implementation of “smart” *in vivo* approaches to toxicity testing that can effectively assess safety for humans and comply with societal expectations for reduction in animal use. A major obstacle in reducing the animals required is the time-consuming and complexity of the pathological endpoints used as markers of toxicity. Such endpoints are prone to inter-animal variability, subjectivity and require harmonisation between testing sites. As a consequence, large numbers of animals per experimental group are required. To address this issue, we propose the implementation of sophisticated stress response reporter mice that we have developed. These reporter models provide early biomarkers of toxic potential in a highly reproducible manner at single-cell resolution, which can also be measured non-invasively and have been extensively validated in academic research as early biomarkers of stress responses for a wide range of chemicals at human-relevant exposures. In this report, we describe a new and previously generated models in our lab, provide the methodology required for their use and discuss how they have been used to inform on toxic risk (likelihood of chemical causing an adverse health effect). We propose our *in vivo* approach is more informative (refinement) and reduces the animal use (reduction) compared to traditional toxicity testing. These models could be incorporated into tiered toxicity testing and used in combination with *in vitro* assays to generate quantitative adverse outcome pathways and inform on toxic potential.

## Abbreviations

AhR: aryl hydrocarbon receptor

hCG: human chorionic gonadotropin

iAs: inorganic arsenic

NBF: neutral-buffered formalin

OECD: Organisation for Economic Co-operation and Development

PBS: phosphate-buffered saline

PFA: para-formaldehyde

TCDD: Tetrachlorodibenzo-p-dioxin

2-MCPD: 2-Monochloropropane-1,3-diol

3-MCPD: 3-monochloropropane-1,2-diol

## Introduction

Development of new manmade chemicals has underpinned an enormous rise in quality of life in areas such as pharmaceuticals, technology, or food production. ^1^ However, humans and the environment need to be protected from potential short/long-term toxic consequences of exposure to these agents and their derivatives. Regulatory procedures ensure appropriate risk assessments are conducted on novel agents before approval for commercial use. In the absence of human data, studies with multi-cellular experimental models are required to detect and estimate toxic potential. *In vivo* models, although with limitations, provide the closest estimation of recommended levels of safe exposure in a physiological context. The implementation of such tests must be appropriate, responsible, and humane, by reducing the numbers of animals in a study, refinement of measurements and implementation of less invasive endpoints. ^2^ This is achieved by continuous application of the 3Rs principles, particularly when vertebrate models such as rodents are being used and is reflected in OECD guidelines updates. For example, in acute inhalation studies, death as an endpoint was substituted for clinical signs of toxicity such as hypoactivity, bodyweight loss, and irregular respiration. ^3^ However, clinical signs often reflect major disturbances of homeostasis, which are very unlikely to occur at relevant human exposure levels and do not inform on the mechanisms involved. Additionally, these tests are prone to subjectivity, inter-animal variability, require harmonisation between testing sites and need large numbers of mice for prolonged periods. For example, the OECD-451 guideline for carcinogenic analysis recommends studies lasting for two years and groups of up to 50 animals for each testing condition. ^4^

New approaches are being proposed to reduce reliance on animal testing for regulatory or research purposes. Although progress has been made, most models still have significant limitations, including the inability to recapitulate the cellular complexity of physiological structures of mammals, or the biochemical and physiological differences between simple organisms and the pathways which define chemical toxicity. Despite promising efforts to develop organoids, a harmonized model has been difficult to establish due to issues such as marked cell variability, aberrant activation of signalling pathways and the lack of physiological complexity. ^5^ For example, metabolic activation of some compounds can initiate toxicity in a different distal tissue. Also, invertebrates do not provide an alternative to higher organisms because of differences in the pathways that define the mode of action as well as the sensitivity of target cells to chemicals. For example, vertebrates are sensitive to the effects of non-genotoxic carcinogens such as TCDD. However, invertebrates are insensitive, consistent with vertebrates possessing an AhR that binds TCDD-like chemicals, whereas invertebrate AhR homologs do not. ^6^ Consequently, the use of complex organisms such as rodents remains a recommended approach for toxicity testing.

**Figure 1.**
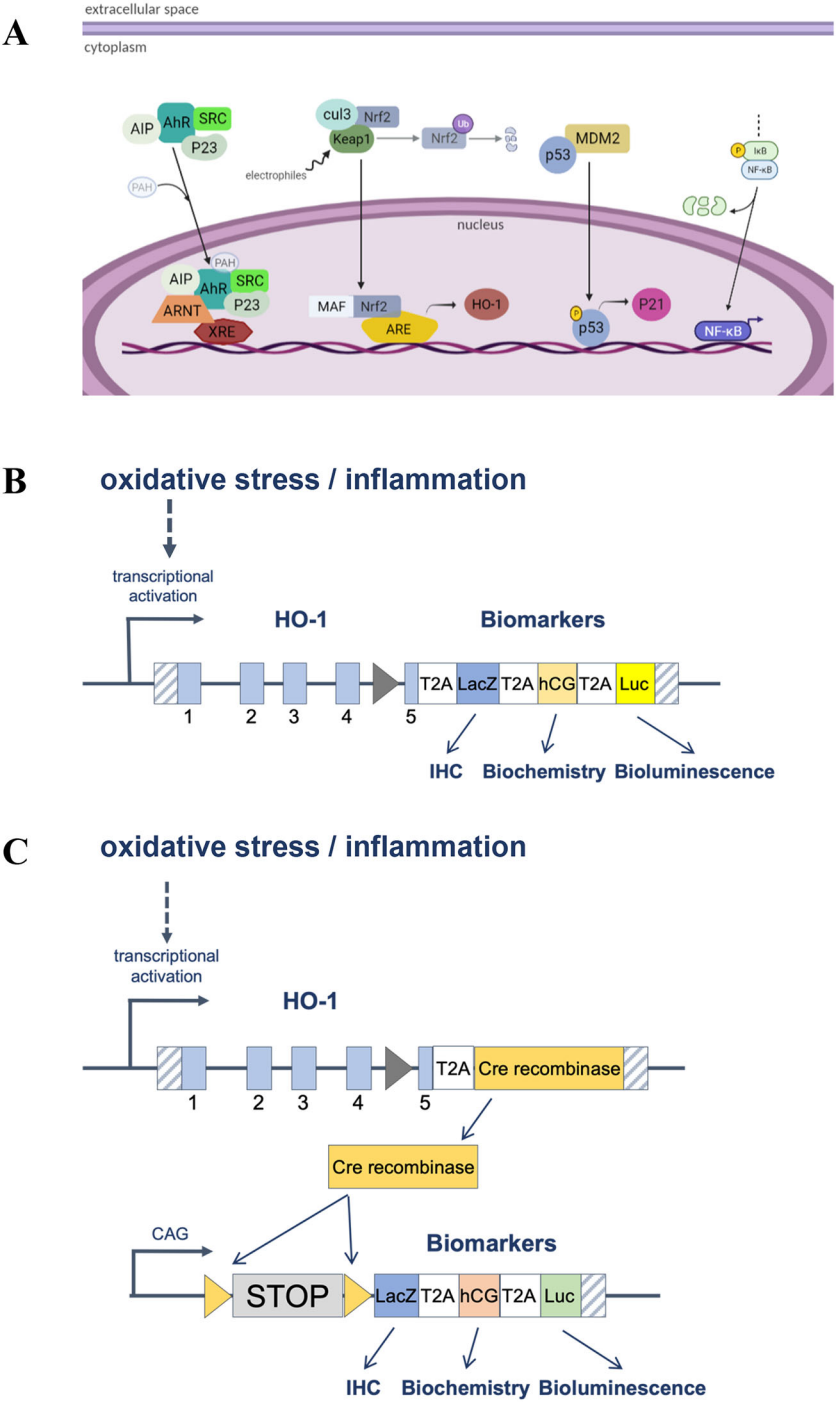
A. Key stress cellular pathways activated by toxicants where reporters have been developed. B and C. Schematic representation of how direct stress reporters (A) and stress shadowing reporters (B) work at the molecular level.

To overcome these challenges, the introduction of new methodologies for conducting a tiered approach “smart” *in vivo* studies with the same level of risk confidence has been proposed. ^7^ Consistent with this paradigm, in this communication we propose the incorporation of stress reporter mice into such tier 3 non-conventional *in vivo* toxicity testing systems ( Table 1). We describe how stress reporter mice can overcome some of the issues surrounding traditional *in vivo* toxicity studies, accelerate toxicity testing and aid in risk assessment while simultaneously reducing animal use. The rationale is that cells are protected from toxic stress through the induction of specific cytoprotective genes ( Figure 1A). Activation of these reporters provides an early biomarker of toxic potential and provides mechanistic information with minimal inter-animal variability. Such models also provide a refined, highly sensitive method of relating dose to toxic effects, a prerequisite for risk assessment. At the molecular level, reporter mice generate easily measurable endpoints in a manner reflecting transcriptional activation of different endogenous genes ( Figure 1B). For example, the *p21* gene is rapidly activated in response to DNA damage. Therefore, *p21* transcriptional activation provides excellent biomarkers of DNA toxic stress. These models carry multiple complementary reporters, including luciferase (non-invasive imaging) as well as β-galactosidase (post-mortem, fine-scale spatial mapping of stressed cells by histochemistry). Moreover, the Hmox1 model expresses hCG, a secreted marker that can be detected in blood. Molecular details for the generation of these lines are described elsewhere ( Table 1). We also present a new stress reporter model, HMOX1 stress shadowing mice, where cells exposed to stress are permanently marked and their subsequent fate is tracked ( Figure 1C).

**Table 1.** Stress reporter models generated in our lab. References to the original publications and subsequent use in toxicologic studies are provided.

Reporter name	Gene	Transcriptional regulation	Toxicity mechanism
HOTT (HMOX1 triple transgenic); NRF2-KO-HOTT ^8^^,^^11^^,^^14^^,^^15^	Hmox1; Nfe2l2-Hmox1	Nrf2, AP1	Oxidative stress; Inflammation
Hmox1_KI_Cre; ROSA26 multireporter	Hmox1	NRF2, AP1	Oxidative stress; Inflammation (stress shadowing)
p21 ^11^^,^^17^	Cdkn1a	P53	DNA damage
CYP1A1_KI_Cre; ROSA26 multireporter ^19^^,^^20^	Cyp1a1	AhR	Xenobiotic metabolism (stress shadowing)
Dual specificity protein phosphatase 1	Dusp1	MAPK3/1	Inflammation and apoptosis
P1ROZ ^16^	hGstp	NRF2, AP1	Oxidative stress, Inflammation
Aldo-keto reductase 1C1	hAkr1c1 promotor	NRF2	Oxidative stress
Interleukin-6	Il-6	NFkB/AP1	Inflammation
CYP2B6-LacZ ^18^	hCYP2B6	CAR/PXR	Xenobiotic metabolism

## Methodology

### Animal husbandry

All animals used in our studies were bred and maintained at the University of Dundee. Mice were housed in open-top cages in temperature-controlled rooms at 21°C, with 45-65% relative humidity and 12 h/12 h light/dark cycle. Mice had *ad libitum* access to food and water. Animals were subjected to standard health and welfare monitoring (twice daily). Environmental enrichment was provided. All animal work was performed under appropriate ASPEL licencing and approved by the Welfare and Ethical use of Animals Committee of the University of Dundee. Experimental design was undertaken following the 3Rs principles and according to the ARRIVE guidelines.

### Study design

Data was obtained using either male or female mice, unless specific sex sensitivity was detected. Heterozygous and homozygous reporter mice are viable and fertile. Mice were 10–18 weeks old, heterozygous for the reporter allele, littermates or age-matched (within three weeks). Animals were randomly assigned to treatment groups. The number of mice per group was determined using the G*power calculator. For bioluminescence and hCG expression, previous work confirmed normal distribution of data using a Kolmogorov-Smirnoff test with standard deviation of 0.35. Sample size required to detect a two-fold difference between groups was n=4, using a standard power of 80% and a type I error of 5%. The changes in reporter activation are highly reproducible, and therefore we are confident 4 animals/group is sufficient to avoid Type I and II errors. We acknowledge that where additional end points are to be measured simultaneously, this may be required to use a higher number of mice. To compare the expression levels between two different groups a t-test was run. Scientists were aware of the nature of the compounds been administered. Analysts can be blinded to sample identity, as was done in our study.

### Tissue harvesting and processing for cryo-sectioning

Tissues were rapidly harvested *post mortem* and fixed for 2 h in 4% PFA (brain, small intestine, skin), 3 h in 10% NBF (liver) or for 18 h in Mirsky’s fixative (other tissues). Tissues were dehydrated for at least 24 h in 30% (w/v) sucrose/PBS buffer at 4°C. Organs were later embedded in Shandon M-1 Embedding Matrix (Thermo Fisher Scientific) in a dry ice-isopentane bath. Sectioning was performed on an OFT5000 cryostat (Bright Instrument Co.). Except for lung (14 μm) and brain (20 μm), all sections were cut at 10 μm thickness (all reagents from SIGMA Aldrich [Merck], unless otherwise indicated).

### *In situ* β-galactosidase staining and histochemistry

Sections were rehydrated in PBS at room temperature (15 min) before incubation overnight at 37°C in X-gal staining solution: phosphate buffer solution (PBS) (pH 7.4) containing 2 mM MgCl _2_, 0.01% (w/v) sodium deoxycholate, 0.02% (v/v) Igepal-CA630, 5 mM potassium ferricyanide, 5 mM potassium ferrocyanide and 1mg/ml 5-bromo-4-chloro-3-indolyl β-D-galactopyranoside (Thermo Fisher Scientific). The next day, slides were washed in PBS, counterstained in Nuclear FastRed (Vector Laboratories) (4 min), washed twice in distilled water (2 min) and dehydrated through 70% and 95% ethanol (4.5 and 1 min respectively) before incubation in Histoclear (VWR) (3 min), air-dried and mounted in DPX mountant (all reagents from Sigma Aldrich, except otherwise indicated).

*In vivo* luciferase imaging and hCG measurement were performed as described before. ^8^

## Results

We exemplify the utility of our reporter models to address different toxicology scenarios using previously published studies. We describe for first time the HMOX1 “stress shadowing” model, that can be applied to the study of chemical exposure toxicity during development and effects in later life.

### Case study 1. Chemicals in the environment

**Figure 2.**
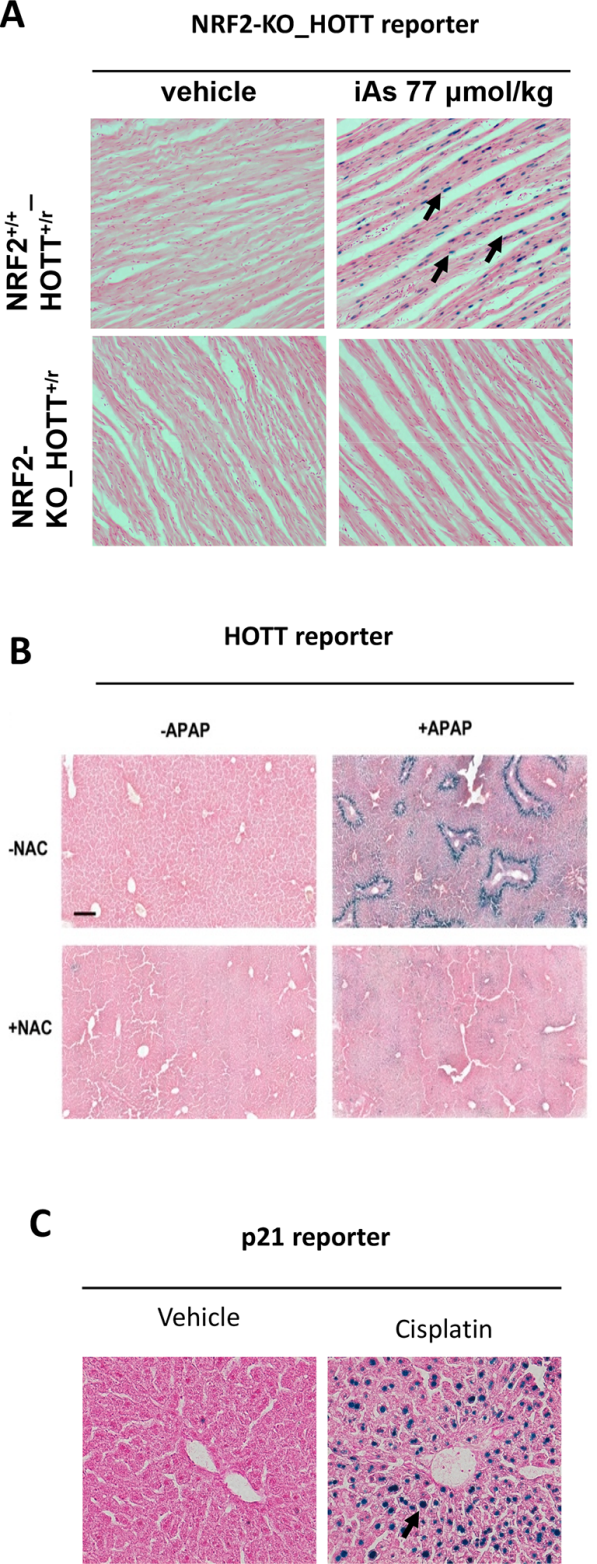
Demonstration of the utility of stress reporter mice to define mechanisms of toxicity. A. NRF2_Hmox1 reporter mice. ^11^ iAs, a naturally occurring metalloid, was dosed at 10 mg/kg, p.o. and 24 h heart tissue (20×) was processed for LacZ staining. B. Hmox1 reporter model. ^8^ Acetaminophen (APAP; in excess, such as an overdose, results in rapid depletion of glutathione within the liver), 300 mg/kg p.o.; NAC (N-acetylcysteine), 300 mg/kg; LacZ staining in liver tissue (10×). C. p21 reporter model was tested with the anti-cancer drug Cisplatin 10mg/kg, which causes damage to nuclear and mitochondrial DNA. ^17^ LacZ staining in liver tissue (10×).

iAs) is present in drinking water and polluted air exposing millions of people. ^9^ Epidemiological studies have linked iAs exposure to the development of numerous diseases including cognitive impairment, cardiovascular failure and cancer. One limitation in the study of iAs toxicity is that the concentrations required to identify potential toxic effects on mouse physiology exceed realistic environmental exposure levels. ^10^ The Hmox1 reporter model provides a robust *in vivo* approach to detect iAs-associated toxicity in a cell- and tissue-specific manner, and, importantly from an NC3Rs point of view, prior to any overt toxic phenotype. In a series of dose- and time-response studies we identified tissue-specific HOTT reporter activation at environmentally relevant concentrations in studies that lasted a maximum of four weeks. Moreover, we demonstrated that oxidative stress is the major *in vivo* mechanism of iAs toxicity using pharmacological (antioxidant N-acetylcysteine) and *in vivo* genetic approaches ( Figure 2A). This demonstrates how the models can also be used to rapidly test treatments of disease. ^11^ Similarly, we have used these reporter models to understand the toxicity of additional environmental chemicals, including cadmium (a nephro- and hepato-toxin found in cigarette smoke), “forever” chemicals and diesel emissions (Inesta-Vaquera, manuscripts in preparation). The information generated can be used in risk assessments and inform policy for health interventions.

### Case study 2. Chemicals in consumer products

2-MCPD and its isomer 3-MCPD are formed during the refinement of vegetable oils in processed foods. 3-MCPD has been classified as non-genotoxic, and possibly carcinogenic, to humans (category 2B; IARC, 2012). In a conventional two-year long feeding study in rats (group size=50), 3-MCPD-induced nephrotoxicity and testicular toxicity. Moreover, an increased incidence for Leydig cell tumours and kidney tubular adenomas was observed. ^12^ The mechanism of 3-MCPD toxicity was not determined, although studies suggested that induction of oxidative stress was involved. ^13^ A shorter (28-day) study using the HOTT model (only five animals per group), reported nephrotoxicity, testicular and liver toxicity. Strikingly, these studies identified a previously unreported induction of oxidative stress in the brain. ^14^^,^^15^ Importantly, the effects of 2-MCPD, which has not been studied in carcinogenicity studies, were less pronounced, suggesting that the molecular toxicity mechanisms of 2-MCPD and 3-MCPD are potentially different. ^14^^,^^15^ A similar approach was used to demonstrate the capacity of the food preservative ethoxyquin to induce liver toxicity, using a combination of our hGSTP1 and NRF2-nulled reporter mice. ^16^

### Case study 3. Drug-induced toxicity

The safety of new therapeutics is paramount in the drug development process. Many drugs targeting the DNA damage response currently developed as anti-cancer therapies have the potential to increase toxicity to normal tissues. The use of reporter mice can accelerate the drug development process by determining which agents (and/or their combinations) possess a stronger toxic potential ( Figure 2B and C). We have determined the capacity of several DNA-damaging agents (ionizing radiation, cisplatin, etoposide) to induce DNA damage in tissues using reporter mice. ^17^ We also illustrated the power of this approach to evaluate the safety of new therapeutic regimens involving combination therapies. We demonstrated that a single exposure to olaparib, a PARP inhibitor licenced for the treatment of a number of cancers, caused DNA damage in mucosal cells lining the mouse large intestine, which was exacerbated in this organ, and the kidney by co-administration of ionizing radiation, suggesting that olaparib has carcinogenic potential.

Drug metabolism is a major step in drug development and pharmacology. Humanised models of drug disposition are described in an accompanying report (see Henderson *et al.* ^16^). The reporter technology can also be applied to identify activators of transcription factors responsible for induction of hepatic drug metabolising enzymes. For example, the constitutive androstane receptor (CAR) activates genes involved in drug metabolism, lipid homeostasis, and cell proliferation. We have developed a humanised rodent model of hCAR/hPXR containing a CYP2B6-LacZ transgene that allows the screening of large numbers of compounds, and studied the capacity of phenobarbital to activate this pathway *in vivo.* ^18^

### Stress shadowing technology

**Figure 3.**
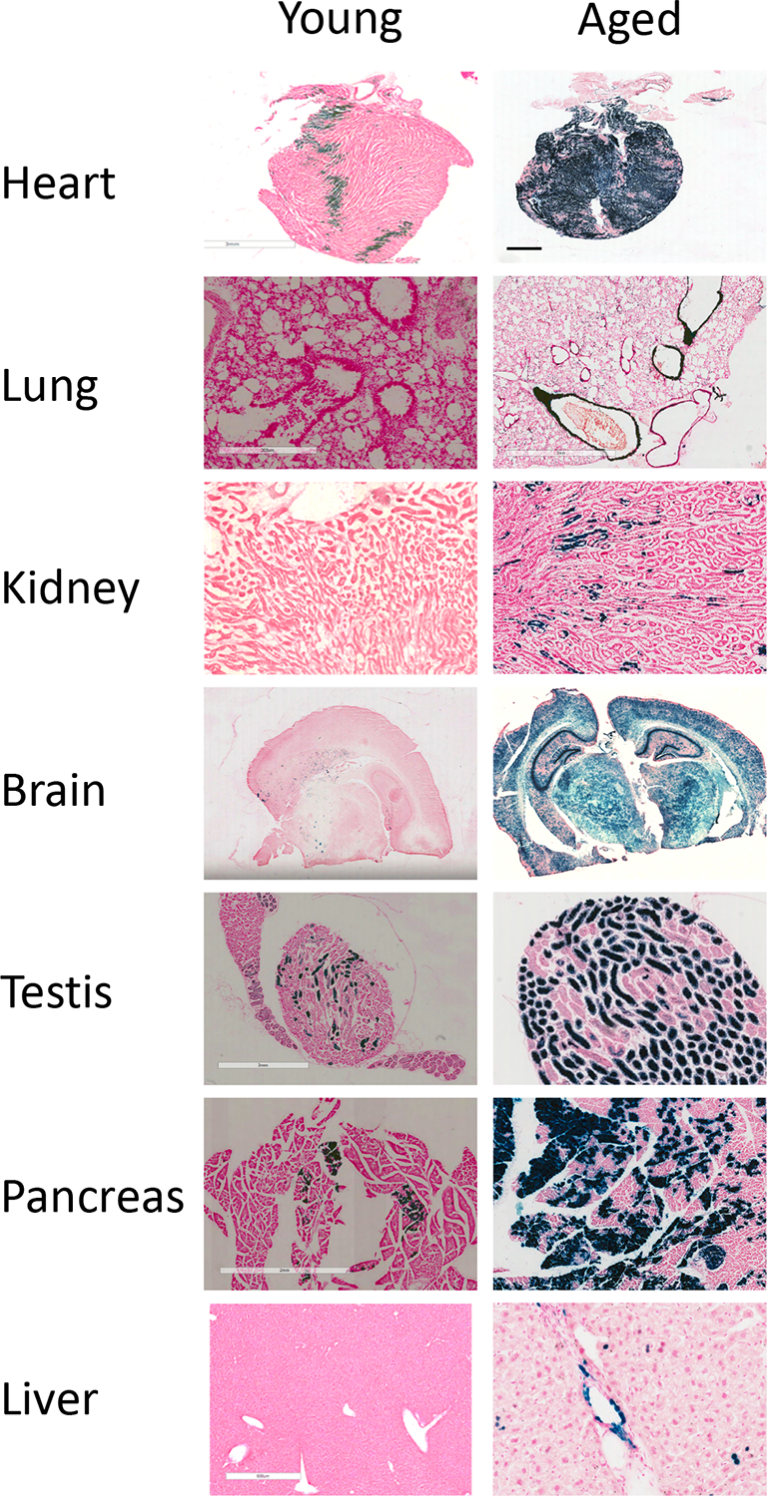
Application of the Hmox1 stress shadowing reporter mice to track metabolic changes during ageing (triplicate mice; young = 9 weeks old; aged = 24 weeks old). LacZ staining performed in indicated tissues.

We report a novel Hmox1 stress-shadowing model. Stress-shadowing is a fate-mapping approach where Cre is driven from environmentally responsive rather than lineage-restricted genes. We engineered a new ‘driver’ mouse strain in which Cre is expressed from the Hmox1 locus (Hmox1 ^Cre^). We also generated a new reporter/tracer strain ROSA26 ^LSL-LacZ-T2A-Luc^ in which a floxed silencing element (Lox-STOP-Lox, LSL) prevents expression of a dual reporter element harbouring both LacZ and luciferase. By crossing these ‘driver’ and ‘tracer’ strains, we generated the HMOX1 stress-shadowing mouse. Stress-induced expression of *Hmox1* lead to Cre expression and, consequently, excision of the floxed silencing element. This permanently switches on the reporter, providing an enduring molecular mark that can be read at any subsequent time point ( Figure 1C). We present a study confirming a low expression of the reporter in tissues of untreated young mice (six weeks old). Interestingly, the reporter becomes activated at older ages in untreated animals, especially in long-lived cells such as cardiomyocytes, neurons, and smooth-muscle cells ( Figure 3). We have confirmed that activation is dependent on Cre and there is no ‘leakiness’ of reporter expression in the ROSA26 ^LSL-LacZ-T2A-Luc^ line (data not shown, but available on request). Therefore, this line can track post-natally the fate of cells exposed during gestation to chemicals that induce Hmox1. This approach was successfully validated in another of our lines where Cre recombinase is driven from the endogenous *Cyp1a1* locus, an AhR-responsive gene. ^19^

## Discussion

We propose the use of stress reporter models to reduce and refine the use of rodents in toxicity testing. We have demonstrated that reporter expression is a reproducible approach to quantify the potential toxic effects of a test substance, avoiding the variability of clinical scoring in conventional studies. Groups of only four animals are required to detect robust changes in luciferase/hCG expression differences between two treatments. Moreover, in our experience, groups of just three mice are sufficient to obtain reproducible results when measuring ß-galactosidase reporter expression as a qualitative endpoint (absence or presence of signal). This is achieved due to stable and specific reporter expression, which can also capture transient metabolic changes difficult to observe by other methods such as transcriptional profiling, as is the case with HO1.

The reporter approach offers a higher sensitivity in detecting homeostasis disturbance compared to traditional clinical signs of toxicity, paving the way to extensive study refinement. For example, test compound concentrations can be at relevant environmental/clinical exposure scenarios, below doses inducing overall toxicity. Similarly, study durations can be significantly reduced. These *in vivo* reporters can therefore be used as early toxicity biomarkers for quantitative risk assessment on test substances by detecting effects at lower levels of exposure, assessing biological effects and providing valuable information on toxicity-associated mechanisms. Moreover, the ubiquitous distribution of these reporters ensures that relevant adverse effects in tissues not directly exposed to the test substance are not missed. Importantly, the assays required to measure reporter expression are inexpensive, fast, reliable and obviate the need for sophisticated laboratory equipment.

The application of reporter mice could provide critical information about potential toxicity in the drug discovery process to avoid unnecessary traditional toxicity studies, and/or initiate studies to establish whether such changes may be of clinical relevance. However, the interpretation of cellular responses observed using these reporters need careful considerations. Cellular responses to toxic agents activate adaptive responses that protect cells from cell death. However, when these pathways are overcome toxicity results. Therefore, the activation of these pathways does not necessarily correlate with toxicity, but are indicative of toxic insults. For example, cisplatin can activate the DNA-damage reporter in liver, but it does not cause hepatotoxicity. The nature of the study will decide the next step in toxicity studies and will still depend on what murine parameters are present. We propose that the generation of stress reporter models on a humanized genetic background will help to overcome some of the potential caveats related to species differences in drug and foreign compound metabolism. For example, in drug toxicity, testing the incorporation of stress reporters into models humanized for metabolic enzymes (e.g. P450s) may further increase the predictive power of in vivo studies in relation to risk to man.

## Data availability

### Underlying data

figshare: F1000R-NC3R Publication - Potential of in vivo stress reporter models to reduce animal use and provide mechanistic insights in toxicity studies, https://doi.org/10.6084/m9.figshare.c.6049598.v2. ^21^

### Reporting guidelines

figshare: ARRIVE Essential 10 Checklist for “Potential of *in vivo* stress reporter models to reduce animal use and provide mechanistic insights in toxicity studies”, https://doi.org/10.6084/m9.figshare.20528352.v1. ^22^

Data are available under the terms of the Creative Commons Attribution 4.0 International license (CC-BY 4.0).

## References

[1] Oeppen J Vaupel JW : Demography. Broken limits to life expectancy. *Science.* 2002;296(5570):1029–1031. 10.1126/science.1069675 12004104

[2] Fischer I Milton C Wallace H : Toxicity testing is evolving! *Toxicol Res (Camb).* 2020;9(2):67–80. 10.1093/toxres/tfaa011 32440338 PMC7233318

[3] Sewell F : A global initiative to refine acute inhalation studies through the use of ‘evident toxicity’ as an endpoint: Towards adoption of the fixed concentration procedure. *Regul. Toxicol. Pharmacol.* 2015;73(3):770–779. 10.1016/j.yrtph.2015.10.018 26505531

[4] Felter SP : Hazard identification, classification, and risk assessment of carcinogens: too much or too little? - Report of an ECETOC workshop. *Crit. Rev. Toxicol.* 2020;50(1):72–95. 10.1080/10408444.2020.1727843 32133908

[5] Parish ST : An evaluation framework for new approach methodologies (NAMs) for human health safety assessment. *Regul. Toxicol. Pharmacol.* 2020;112:104592. 10.1016/j.yrtph.2020.104592 32017962

[6] Ankley GT : Adverse outcome pathways: a conceptual framework to support ecotoxicology research and risk assessment. *Environ. Toxicol. Chem.* 2010;29(3):730–741. 10.1002/etc.34 20821501

[7] Ball N : A framework for chemical safety assessment incorporating new approach methodologies within REACH. *Arch. Toxicol.* 2022;96(3):743–766. 10.1007/s00204-021-03215-9 35103819 PMC8850243

[8] McMahon M : Measuring in vivo responses to endogenous and exogenous oxidative stress using a novel haem oxygenase 1 reporter mouse. *J. Physiol.* 2018;596(1):105–127. 10.1113/JP274915 29086419 PMC5746521

[9] Straif K : A review of human carcinogens--Part C: metals, arsenic, dusts, and fibres. *Lancet Oncol.* 2009;10(5):453–454. 10.1016/S1470-2045(09)70134-2 19418618

[10] Tokar EJ : Cancer in experimental animals exposed to arsenic and arsenic compounds. *Crit. Rev. Toxicol.* 2010;40(10):912–927. 10.3109/10408444.2010.506641 20812815 PMC3076186

[11] Inesta-Vaquera F : Application of the in vivo oxidative stress reporter Hmox1 as mechanistic biomarker of arsenic toxicity. *Environ. Pollut.* 2021;270:116053. 10.1016/j.envpol.2020.116053 33213951

[12] Cho WS : Carcinogenicity study of 3-monochloropropane-1,2-diol in Sprague-Dawley rats. *Food Chem. Toxicol.* 2008;46(9):3172–3177. 10.1016/j.fct.2008.07.003 18680782

[13] Buhrke T : Oxidative inactivation of the endogenous antioxidant protein DJ-1 by the food contaminants 3-MCPD and 2-MCPD. *Arch. Toxicol.* 2018;92(1):289–299. 10.1007/s00204-017-2027-5 28707023

[14] Schultrich K : Effects of 2-MCPD on oxidative stress in different organs of male mice. *Food Chem. Toxicol.* 2020;142:111459. 10.1016/j.fct.2020.111459 32474023

[15] Schultrich K : Correlation between 3-MCPD-induced organ toxicity and oxidative stress response in male mice. *Food Chem. Toxicol.* 2020;136:110957. 10.1016/j.fct.2019.110957 31712104

[16] Henderson CJ McLaren AW Wolf CR : In vivo regulation of human glutathione transferase GSTP by chemopreventive agents. *Cancer Res.* 2014;74(16):4378–4387. 10.1158/0008-5472.CAN-14-0792 24934809 PMC4134684

[17] McMahon M : Olaparib, Monotherapy or with Ionizing Radiation, Exacerbates DNA Damage in Normal Tissues: Insights from a New p21 Reporter Mouse. *Mol. Cancer Res.* 2016;14(12):1195–1203. 10.1158/1541-7786.MCR-16-0108 27604276 PMC5136472

[18] McMahon M : Constitutive androstane receptor 1 is constitutively bound to chromatin and ‘primed’ for transactivation in hepatocytes. *Mol. Pharmacol.* 2019;95(1):97–105. 10.1124/mol.118.113555 30361333 PMC6277922

[19] Schiering C : Feedback control of AHR signalling regulates intestinal immunity. *Nature.* 2017;542(7640):242–245. 10.1038/nature21080 28146477 PMC5302159

[20] Villa M : Aryl hydrocarbon receptor is required for optimal B-cell proliferation. *EMBO J.* 2017;36(1):116–128. 10.15252/embj.201695027 27875245 PMC5210087

[21] Inesta F : F1000R-NC3R Publication - Potential of in vivo stress reporter models to reduce animal use and provide mechanistic insights in toxicity studies. figshare. [Dataset]. Collection. 2022. 10.6084/m9.figshare.c.6049598.v2 PMC10329194 37427015

[22] Inesta F : ARRIVE guidelines checklist - F1000 report. figshare. Dataset. 2022. 10.6084/m9.figshare.20528352.v1

