# Supplementary material for: Potential of in vivo stress reporter models to reduce animal use and provide mechanistic insights in toxicity studies
Source: F1000Res. 2023 Aug 10;11:1164. Originally published 2022 Oct 11. [Version 2] doi: 10.12688/f1000research.123077.2 (PMC10329194; doi:10.12688/f1000research.123077.2)
Supplement: Peer review history

# Peer review: Reviewer response for version 2

**DOI:** 10.5256/f1000research.153834.r195616

**Published:** 2023-08-18

**Authors:** Mark Miller

**Affiliations:** 1 University/BHF Centre for Cardiovascular Sciences, University of Edinburgh, Edinburgh, UK

I have no further comments.

Is the work clearly and accurately presented and does it cite the current literature?

Yes

If applicable, is the statistical analysis and its interpretation appropriate?

Partly

Are all the source data underlying the results available to ensure full reproducibility?

Yes

Is the study design appropriate and is the work technically sound?

Yes

Are the conclusions drawn adequately supported by the results?

Yes

Are sufficient details of methods and analysis provided to allow replication by others?

Partly

Reviewer Expertise:

in vivo rodent models, air pollution, cardiovascular disease

I confirm that I have read this submission and believe that I have an appropriate level of expertise to confirm that it is of an acceptable scientific standard.

# Peer review: Reviewer response for version 1

**DOI:** 10.5256/f1000research.135145.r153304

**Published:** 2023-07-07

**Authors:** Heather M. Wallace

**Affiliations:** 1 School of Medicine, Medical Sciences and Nutrition, Institute of Medical Sciences, University of Aberdeen, Aberdeen, UK

This is an interesting and well written overview addressing the the use of in vivo reporter models and how these might provide mechanistic detail not available via other methods. The paper discusses toxic risk and the text would benefit from this being clearly defined.  The use of "smart" approaches is good but is this being used in the well known sense of smart goals? The authors argue that n = 4  is sufficient for the in vivo studies. It would be good to provide the evidence supporting this statement.  It is stated that risk assessment is qualified by biomarkers but this statement needs support and explanation.  

 A minor correction OCDE should be OECD through out.

Is the work clearly and accurately presented and does it cite the current literature?

Yes

If applicable, is the statistical analysis and its interpretation appropriate?

Not applicable

Are all the source data underlying the results available to ensure full reproducibility?

Yes

Is the study design appropriate and is the work technically sound?

Yes

Are the conclusions drawn adequately supported by the results?

Yes

Are sufficient details of methods and analysis provided to allow replication by others?

Yes

Reviewer Expertise:

Toxicology and risk assessment particularly  of contaminants

I confirm that I have read this submission and believe that I have an appropriate level of expertise to confirm that it is of an acceptable scientific standard.

# Peer review: Reviewer response for version 1

**DOI:** 10.5256/f1000research.135145.r174523

**Published:** 2023-06-06

**Authors:** Mark Miller

**Affiliations:** 1 University/BHF Centre for Cardiovascular Sciences, University of Edinburgh, Edinburgh, UK

This is a short review with original data outlining the use of the investigators’ mouse models with reporters of stress pathways. Data is shown demonstrating the presence of the reporter in histological sections from mice treated with various xenobiotics or aged animals for pathways that include oxidative stress and DNA damage, with prevention by anti-oxidants or genetic knockout. The authors conclude that these models could be used to detect activation of key stress pathways at more environmentally relevant exposures of stimulants without inducing overt toxicity, while offering the potential to reduce the length of exposure and number of animals per group. The paper is clearly written and the conclusions are reasonable for the examples shown in the paper.

 Minor comments:

1. Please state the source of the SD=0.35 used for the power calculations. I accept that the biological variability of the reporter expression is very low, however, n=4/group is very low for statistical analysis and risk of type I and II errors. The authors should comment on this. Similarly, the authors may also note that the presence of the reporter will likely be one variable measured in any detailed study using these mice but inevitably other functional and pathological parameters will be measured as well – these will still require higher n-numbers. Nonetheless, experiments with n=4 with reporter expression alone would still have great value as a screening and dose-finding tool.
2. It would be useful to show some quantitative data as well as the qualitative images. In particular, this could be used to demonstrate the variability of the reporter parameter or that more environmentally-relevant doses of stressors can be used, i.e. a dose response curve to a stimulant required to induce the reporter in these mice compared to a typical parameter used assess toxicity in normal mice to demonstrate.
3. If space permits, I would like to see more data from the examples outlined in the narrative text. The authors mention that these models can be used for non-invasive imaging – this data in particular would be good to show.
4. Please add a little further discussion on the physiological relevance of these pathways. For example, playing devil’s advocate, a dose of xenobiotic/drug may stimulate the reporter, however, this could be functionally harmless and it is only from greater/longer exposure that other biological responses become impaired causing a detrimental effect on health. In which case the reporter may give a false impression of the dose of exposure that is harmful, and potentially cause a drug to fail screening despite being promising in terms of risk-benefit in moderated doses.
5. Is there any impact on breeding or known non-specific effect of the reporter compared to the wild-type mice?
6. Ensure that figure legends are fully explained, e.g. describe why APAP and cisplantin are used – many readers will not be familiar with the type of stress/toxicity caused by these agents.

Is the work clearly and accurately presented and does it cite the current literature?

Yes

If applicable, is the statistical analysis and its interpretation appropriate?

Partly

Are all the source data underlying the results available to ensure full reproducibility?

Yes

Is the study design appropriate and is the work technically sound?

Yes

Are the conclusions drawn adequately supported by the results?

Yes

Are sufficient details of methods and analysis provided to allow replication by others?

Partly

Reviewer Expertise:

in vivo rodent models, air pollution, cardiovascular disease

I confirm that I have read this submission and believe that I have an appropriate level of expertise to confirm that it is of an acceptable scientific standard.
